# Use of Medicines from the Group of Benzodiazepines in the Period of 2003-2013 Year in the Republic of Macedonia

**DOI:** 10.3889/oamjms.2015.004

**Published:** 2014-12-24

**Authors:** Tatjana Petrushevska, Vesna Velik Stefanovska

**Affiliations:** 1 *Ministry of Health, Skopje, Republic of Macedonia*; 2 *Institute for Epidemiology and Medical Biostatistics, Medical Faculty, Ss. Cyril and Methodius University of Skopje, Skopje, Republic of Macedonia*

**Keywords:** benzodiazepines, drug dependence, diazepam, alprazolam, drug consumption

## Abstract

**AIM:**

The aim of this study was to analyze the use of benzodiazepines in the period of 2003-2013 year in the Republic of Macedonia (MKD).

**METHOD:**

The study was cross sectional and analyzes the available data on the use of benzodiazepines in the country. This study used several sources of data: Statistical Reports of the United Nations regarding the consumption of psychotropic substances; data from the Ministry of Health associated with the use of benzodiazepines (BZD), derived from reports of Ministry of Health stakeholders; Data extracted from the database of the Ministry of Health in the electronic database “My term” and Analysis of reports of Health Insurance Fund.

**RESULTS:**

The analysis for the period 2003-2013 showed that the most consumed drug in MKD from the group of BZD is Diazepam, with 54.8 and predominantly is use of diazepam of 5 mg with 59 %. According to the Health Insurance Fund, Diazepam is second most prescribed medicaments of all medicaments with marketing authorization in MKD.

**CONCLUSIONS:**

The analysis shows that the use of BZD in MKD is particularly high. Limited number of studies was performed for this kind of drugs relating to their effects; differences in use between genders; adult population. There is need for additional focused research that will contribute to developing a full picture of the situation.

## Introduction

Benzodiazepines are used to treat anxiety, insomnia, seizures, muscle spasms, alcohol withdrawal and as a premedication for medical or dental procedures [1]. Benzodiazepines influence to increase the effect of the neurotransmitter gamma-amino butyric acid (GABA) acts by facilitating the binding of the inhibitory neurotransmitter GABA in different GABA receptors in the CNS, resulting in sedative, hypnotic action, encourages sleep, anxiolytic, anti-anxiety, anticonvulsant and muscle relaxant properties [2]. In the group of benzodiazepines with the United Nations Conventions on drug control [3] 34 substances are classified. Thirty-three BZD are included in the list of Convention IV, and 22 of these are classified as BZD anxiolytics. In the last decade, the total production of these substances ranges from 19.4 billion and 29.9 billion S-DDD statistically calculated Defined Daily Dose [4], calculated per year for the period 2003 to 2011.

In 2012 the highest consumption on a worldwide scale is marked the BZD alprazolam, which covers 29% (4.6 billion S-DDD, the total amount of produced type BZD anxiolytics; Diazepam covers 26% (4.3 billion S-DDD), lorazepam 21% (3.3 billion S-DDD); bromazepam 8% (1.3 billion S-DDD) and others. Between 2003-2012 the largest manufacturers worldwide BZD are placed in China (1.9 billion S-DDD), India and Italy (7 billion S-DDD) [5]. According to the United Nations, Diazepam is still the most used psychotropic substances of the BZD anxiolytics and it is used in all regions of the world for medical purposes and very frequently is observed misuse and diversion to illegal purposes. Though the highest level of diazepam produced worldwide was 113 tons in 2004, according to statistics from the United Nations, the quantity in the following years shows a drastic decline in production, but this medicine is still widely used. Countries that emphasize the most used diazepam in 2012 counted in statistics calculated Defined Daily Dose per 1000 inhabitants are in Ghana (61,5 S-DDD), Croatia (35,8 S-DDD), Macedonia (23,2 S-DDD) and Uruguay (18,9 S-DDD) [4]. In the group of BZD sedative - hypnotics are twelve psychotropic substances, of which MKD marketed flurazepam, midazolam, nitrazepam, temazepam. The biggest producers of this group of substances are USA, Germany, Italy and Switzerland.

To have a complete picture of where does the great medical use of drugs from the group of BZD, and perceived independent, non-medical use of medicines of this type, the paper provides a brief overview of pharmacological effect of these medicines. No medical use and abuse of drugs prescription is present as a general condition in all countries, regardless of interventions, health authorities, health professionals, regulatory institutions. But the phenomenon is the combined prescription of medicines from BZD group and opioid analgesics. There are pharmacokinetic interactions between benzodiazepines and opioid analgesics. Complex pharmacodynamic effects of these drugs vary and their combined effects are able to produce significant respiratory depression [6]. Moreover, considering the existence of an opportunity for self-purchase of drugs in pharmacy, health workers have to inform patients about possible overdose by taking a combination of drugs especially in these categories [6]. Use of this group of drugs for a long-time shows that orally taken BZD without additionally consumed substances (such as other drugs, alcohol, etc.), can rarely cause significant morbidity or mortality. But in mixed overdoses, they may potentiate the effect of alcohol or other sedative hypnotics. BZD overdose often occurs in association with other substances. In 2011, a total of 82,086 BZD exposure as the single substance reported to the U.S. Centers for control of poisoning, 300 (0.004%) resulted in major toxicity and 15 (0.0001%) resulted in death [7]. The World Health Organization suggests that drug interactions are a leading cause of morbidity and mortality [8].

We can’t neglect the data imply that drugs of the benzodiazepine group extensively used by people who have developed a drug addiction. On this topic are conducted numerous researches aimed to determine the reasons for the use of BZD by these individuals, whether the reason is the existence of psychiatric co-morbidity and/or only to supplement the amount of opiates/opioids is of interest to implement additional medical stream. The scientific research conducted, demonstrated that most patients addicted to drugs have additional, besides reliability, at least one severely impaired health disorder - psychiatric co-morbidity. The study conducted in Baltimore, USA, during the three-year analysis of patients on treatment for drug addiction showed that 60.6% of patients with dependence on psychoactive substances are present in psychiatric co- morbidity, and more than 30% have at least two psychiatric disorders [9]. Studies show higher rates of psychiatric co-morbidity in people who are dependent on cannabis (76%) in compared addiction with heroin and cocaine (60%). The rate of use of benzodiazepines is high among persons with severe mental illness and co-morbidity of drug addiction, as well as in patients with severe mental illness, only [10].

Members of the females appear significantly more psychiatric disorder, compared with members of the male sex (73.7% in comparison with 55.4%) [11]. There was an association between depressive symptoms with worse patient outcomes from treatment, suggesting the need for attention to depressive symptoms and subjective distress in order better clinical approach to the treatment of substance abuse and consequently improve the results of treatment [12]. As a result, many patients with opioid dependence require treatment with psychotropic drugs, also. Great use and abuse of anxiolytic drugs, benzodiazepines and sedative-hypnotics by persons with opioid dependence who are on substitution treatment is perceived phenomenon.

Concerns for the use of BZD together with substitution therapy for the treatment of drug addiction are coming from the mechanism of action of these drugs. The opioids, including methadone, inhibit the action of breathing through medullar part receptors, the respiratory center. Benzodiazepines (and also alcohol is often used in combination) act synergistically through these mechanisms of action and this may explain the possibility of fatal overdose in the presence of opioids and benzodiazepines and / or alcohol [13]. Studies show that the most commonly used benzodiazepine - diazepam is not associated with a change of methadone concentrations in plasma, but reported significant pharmacodynamic interactions between diazepam and methadone and diazepam and buprenorphine [14-15]. Diazepam is associated with increased sedation, and weakened performance in conducting psychological tests. On the other hand the fatal consequences may occur with concomitant use of methadone and alprazolam (also BZD, which last period is used often) [16], where the concentration of methadone in the blood confirmed findings in the toxic range, suggests that the pharmacodynamic interaction between methadone and alprazolam emphasize toxicity [17-18]. Morbidity and mortality that is associated with co-ingestion of methadone or buprenorphine and BZD suggests that caution should be particularly cautious in prescribing benzodiazepines for those receiving methadone or buprenorphine treatment of opioid dependence, and that doctors should follow indications related disorders arising from BZD abuse and need to treat these patients appropriately. Pharmacodynamic interactions especially when two or more drugs which are able to produce similar pharmacological effects are taken at the same time, can result in significant adverse effects, such as buprenorphine and alprazolam inject together resulting deaths are thought to be associated with depression central nervous system (CNS) and reduced breathing (fatal respiratory depression) [19]. There is a significant rate of relaps of taking BZD after a certain period in long-term users of BZD [20].

Sedatives such as benzodiazepines are the most commonly prescribed drugs for the treatment of insomnia in people addicted to drugs, but also to most other people. Benzodiazepine use is associated with patient characteristics, mostly female, with the use of analgesics and anti-depressants, and the presence of cancer in particular or chronic heart failure [21].

This paper aims to point out the trend of the use of BZD nationwide for over 10 years with special emphasis on the type, quantity and proportion of drugs issued by the group of BZD prescription versus total amounts of benzodiazepines used in the country.

## Materials and Methods

The research presents an analytical cross-sectional study conducted from January to March 2014. The study analyzes the available data on the use of benzodiazepines in the country. This study used several sources of data: Statistical Reports of the United Nations regarding the consumption of psychotropic substances of all countries in the world including MKD; Data from the Ministry of Health, associated with use of drugs from the group of benzodiazepines (BZD), derived from reports of stakeholders in MKD in format for reporting to the United Nations, the coverage period is 2003-2013; Data extracted from the database of the Ministry of Health on the basis of MKD prescribed and implemented electronic prescriptions registered in database “My term” period of coverage June 2013 - May 2014; analysis reports of the Health Insurance Fund from the period 2008-2013.

### Statistical processing

Descriptive statistical method was applied for processing quantitative data. The data obtained from the survey were processed in Excel (data analysis), version 10, and are presented in tables and graphics.

## Results

The analysis showed that, in the country, over a period of 10 years, from a group of benzodiazepines, with different dynamics expressed in popularity and use, the following psychotropic substances are circulating: Alprazolam, Bromazepam, Diazepam Klorazepat, Flurazepam, Lorazepam, Medazepam, Midazolam, Nitrazepam, Prazepam, Tetrazepam.

In accordance with Anatomical and Therapeutic Classification of medicines (ATC) introduced by the World Health Organization, BZD are classified in: first group N05BA, which implies N nervous system, N05 psyholeptics, N05B ansyolitics, N05BA benzodiazepine derivatives. This group includes: diazepam N05BA01 the defined daily dose DDD [22] of 10 mg; bromazepam N05BA08 the defined daily dose of 10 mg; lorazepam N05BA06 the defined daily dose of 2.5 mg, with a daily medazepam N05BA03 defined dose of 20 mg; prazepam N05BA011 the defined daily dose of 30 mg, and group 2 N05S hypnotics and sedatives N05CD benzodiazepin derivatives including flurazepam N05CD01 the defined daily dose of 30 mg; midazolam N05CD08 the defined daily dose of 15 mg; Group three N03BX07muskulni tetrazepam relaxants. Analysis of data from the Ministry of Health associated with the use of drugs from the group of benzodiazepines (BZD), derived from reports of drug agents in MKD format for reporting to the United Nations, the coverage period 2003-2013, points that (Table 1) the most utilized in the MKD is a drug Diazepam, with more than half of all amounts used BZD.

**Table 1:** Summary of quantities used BZD in MKD year period from 2003 to 2013 (kg).

	Aprazolam	Bromazepam	Diazepam	Clorazepate	Flurazepam	Lorazepam	Medazepam	Midazolam	Nitrazepam	Prazepam	Tetrazepam	Total
**2003**	**1**	**1**	**253**	**0**	**4**	**7**	**22**	**4**	**1**	**26**	**0**	**319**

	0,3%	0,3%	79,3%	0,0%	1,3%	2,2%	6,9%	1,3%	0,3%	8,2%	0%	7%

**2004**	**1,5**	**193**	**148**	**2**	**7**	**5**	**10**	**1**	**1**	**24,44**	**0**	**392,94**

	0,4%	49,1	37,7%	0,5%	1,8%	1,3%	2,5%	0,3%	0,3%	6,2%	0,0	8,7%

**2005**	**2,4**	**85**	**173**	**1**	**6,4**	**8**	**17**	**0**	**0,05**	**42**	**0**	**334,85**

	0,7%	25,4%	51,7%	0,3	1,9%	2,4%	5,1%	0,0	0,0	12,5%	0,0	7,4%

**2006**	**3**	**87**	**180**	**0**	**7**	**9**	**10**	**0,5**	**0,1**	**43**	**0**	**339,6**

	0,9%	25,6%	53,0%	0,0	2,1%	2,7%	2,9%	0,1%	0,0	12,7%	0,0	7,5%

**2007**	**5**	**99**	**221**	**0**	**8**	**17**	**6**	**0**	**0,3**	**34**	**0**	**390,3**

	1,3%	25,4%	56,6%	0,0	2,0%	4,4%	1,5%	0,0	0,1%	8,7%	0,0	8,6%

**2008**	**7**	**145**	**286**	**0**	**10,47**	**7**	**17**	**1**	**0,77**	**30**	**0**	**504,5**

	1,4%	28,8%	56,7%	0,0	2,1%	1,4%	3,4%	0,2%	0,2%	5,9%	0,0	11%

**2009**	**8**	**94**	**306**	**0**	**9**	**10**	**13**	**1**	**1**	**50**	**0**	**492**

	1,6%	19,1	62,2%	0,0	1,8%	2,0%	2,6%	0,2%	0,2%	10,2%	0,0	11%

**2010**	**9**	**104**	**103**	**0**	**9**	**9**	**1**	**1**	**0,3**	**47**	**1**	**284,3**

	3,2%	36,6%	36,2%	0,0	3,2%	3,2%	0,4%	0,4%	0,1%	16,5%	0,4	6,3%

**2011**	**8**	**138**	**328**	**0**	**8**	**11**	**12**	**1**	**1**	**38**	**2,5**	**547,5**

	1,5%	25,2%	59,9%	0,0	1,5%	2,0%	2,2%	0,2%	0,2%	6,9%	0,5%	12%

**2012**	**9**	**63**	**165**	**0**	**11**	**23**	**15**	**0,4**	**0,55**	**62**	**2**	**350,95**

	2,6%	18,0%	47,0%	0,0	3,1%	6,6%	4,3%	0,1%	0,2	17,7%	0,6%	7,8%

**2013**	**8**	**193**	**301**	**0**	**5,34**	**3,2**	**8,43**	**0,4**	**0,5**	**22**	**1,16**	**543,03**

	1,5%	35,5%	55,4%	0,0	1,0%	0,6%	1,6%	0,1%	0,1%	4,1%	0,2%	12%

**TOTAL**	**61,9**	**1202**	**2464**	**3**	**85,24**	**95.34**	**131.43**	**10.3**	**6,8**	**418,44**	**6,66**	**4498,9**

	1,4%	26,7%	54,8%	0,1%	1,9%	2,1%	2,9%	0,2%	0,2%	9,3%	0,1%	100%

**Figure 1:**
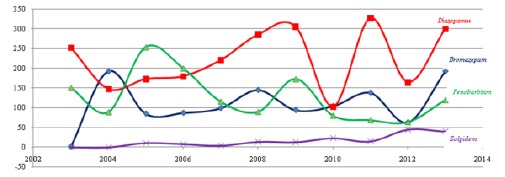
*The use of Diazepam and Bromazepam group of BZD, Phenobarbital group barbiturates and zolpidem group Z-drug period 2003-2013*.

With the emergence of BZD group of drugs, barbiturates because of their greater toxic and dependence predisposition are largely dismissed. Beside that, the drug Phenobarbital representative group of barbiturates is still in use in MKD and worldwide. As part of the analysis is a comparison of three generations of drugs, such as: the use of phenobarbital versus BZD as a group of drugs that eradicate the use of barbiturates group; BZD group and the new group of drugs called non-benzodiazepines or “Z-drugs”, which are developed in order to replace BZD to offer safer treatment for patients with fewer side effects. Non-benzodiazepines are a class of psychoactive drugs that are very similar to benzodiazepines, almost entirely the same as benzodiazepines pharmacodynamics, and completely different chemical structure and therefore not associated with benzodiazepines at the molecular level [23]. These drugs are sedatives and are used exclusively for the treatment of mild insomnia. They are safer than the older barbiturates, in particular in predisposition for overdose; in comparison with benzodiazepines have fewer tendencies to induce physical dependence, although these issues are not yet fully confirmed, as noted side effects like amnesia and rarely hallucinations [24]. The analysis showed that the amount of use of Phenobarbital in MKD is the second most utilized BZD between bromazepam and diazepam first (Figure 1).

Diazepam is convincingly the most commonly used preparation of drugs for the pharmacological indication. A decline is shown in use over the period 2004-2006, when dominance have the barbiturates phenobarbital and oscillations of use is shown in 2010 and 2011.

**Figure 2:**
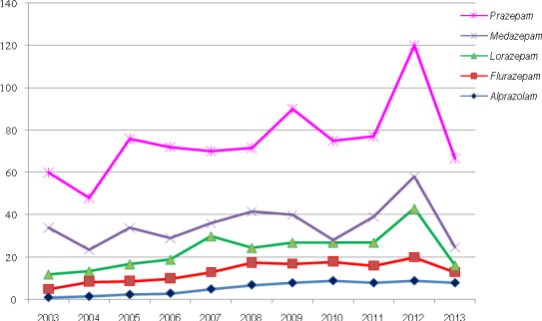
*Trend of use of Prazepam, Medazepam, Lorazepam, Flurazepam, Alprazolam 2003-2013*.

Analysis of the use of BZD shown that despite Diazepam and Bromazepam, Alprazolam, Flurazepam, Lorazepam, Medazepam, Prazepam are significantly used. The ratio of the use of these five BZD during the ten years analyzed is: Prazepam 36%, 11% Medazepam, Lorazepam use 9%, 7% Flurazepam, and 5% use of Alprazolam.

While the use of Flurazepam and Alprazolam is constant, with a slight gradual increase until 2012, use of Lorazepam increased in 2007 by 7.4% as a percentage of use of 8.3% in 2006, and the level of use in 2007 was 15.6%. Then use declines of 6.4% which decreased by 9.2%. It also marks another peak, increased use of Lorazepam in 2012 by 10% and it is 21%. The use of Medazepam seen several oscillation and in 2004 declined the use of 9% and it was 7.6%, then increased in 2005 by 5.3% and amounted to 13% after a slight decline again, following growth use in 2008 and it stands at 12.9%, and in 2010 registered a sharp drop in the use of Medazepam 9%. Oscillation shows the use and re-growth in 2012 when it reached 11.4% of use of BZD. The use of Prazepam is relatively stable and high, 3 observed oscillations and a small decline in the level of 5.6% in 2004, a period of stable continuous level of usage and peak in 2010 to a level of 11.4% in 2009 and 14% in 2012. Noted that all five BZD use declined in 2013 and 2% of Alprazolam, Flurazepam 7%, down from 18% Lorazepam, 5% and 5% Medazepam, Prazepam.

**Figure 3:**
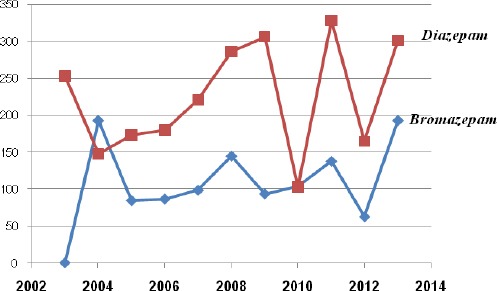
*Trend in the use of diazepam and bromazepam 2003-2013*.

The ratio of use of the two most used BZD in MKD is diazepam 70%, versus 30% bromazepam. The use of diazepam in the analyzed ten years is continuously high, with some oscillations observed: in 2004 decreased by 10% and use level reaches 30%, then gradually to grow by 5% on 2005, then an additional 1.4% in 2006, increased by an additional 8.3% in 2007 and 13% in 2008, to reach high peak of 62% of use in 2009. Then there has been a sharp decline from 40% of use in 2010 and reaching a record low level of use of 20.9%. In 2011 the use marks a big change again this time with an increase of 45.5% and reaching the highest level of use of 66.4%. In 2012 recorded a re-oscillation by reducing the use of 33% to reach the level of use in 2013 of 60.9%. The use of Bromazepam is with oscillations also, but not so strong peaks as in the case of Diazepam. High use in 2004 of 39% dropped to 17% and keeping the use of these scales to more significant growth in 2008 year and achieving a level of 29.4%, then re-drop from 10% in 2009 and growth in 2011 increased by 8.9% and reaching the level of 27.9%, re-oscillation is recorded in 2012 declined the use of 15%, and again increase of 26.3% in 2013 and reaching the level of 39% usage.

In terms of the prescriptions of BZD at the pharmacy, in the analyzed period of 12 months (June 2013-May 2014) time when the electronic database of the Ministry of Health “My term” was introduced, the most prescribed and also released most of the prescription drugs from the group of a BZD are: diazepam 5 mg of 59%, 2mg of 40% and 1% of 10 mg (Table 2).

**Table 2:** Summary of three most prescribed BZD monthly and pharmaceutical ratio of strength versus drug Phenobarbital.

2013							2014					

	June	July	August	September	October	November	December	January	February	March	April	May	Total
bromazepam 1,5 mg	29	1183	2719	3298	3789	3750	4008	3718	3745	3989	4159	2087	36474
bromazepam 3 mg	124	5410	13590	16149	18394	18405	19715	18648	18376	19387	19663	11039	178900
bromazepam 6 mg	20	563	1351	1673	1763	1817	1891	1814	1849	2034	1932	1159	17866
**Total**	173	7156	17660	21120	23946	23972	25614	24180	23970	25410	25754	14285	233240
diazepam 10 mg	1	156	423	528	601	583	578	539	525	585	566	351	5436
diazepam 2 mg	1	5430	17638	22520	25836	26238	27536	26616	26604	27953	28549	15373	250294
diazepam 5 mg	152	10877	28542	34414	38085	37774	40354	39193	38267	40222	41501	22731	372112
**Total**	154	16463	46603	57462	64522	64595	68468	66348	65396	68760	70616	38455	627842
phenobarbital 100 mg	7	795	2641	3051	3395	3434	3614	3418	3385	3418	3434	2038	32630
phenobarbital 15 mg	1	91	113	160	150	146	130	150	155	132	136	50	1414
**Total**	8	886	2754	3211	3545	3580	3744	3568	3540	3550	3570	2088	34044
alprazolam 0,25 mg	84	3736	9443	11539	12823	13328	13921	14044	13783	14667	14958	7610	129936
alprazolam 0,5 mg	123	6041	15687	18306	20313	20396	21930	21398	20942	22210	22511	12096	201953
alprazolam 1 mg	4	316	874	1056	1156	1112	1224	1199	1222	1265	1241	717	11386
**Total**	211	10093	26004	30901	34292	34836	37075	36641	35947	38142	38710	20423	343275

With regard to Bromazepam (Table 2), the most prescribed bromazepam is 3mg of 76%, bromazepam 1.5 mg is 16.6% and bromazepam 6mg is 7.6%. Alprazolam 0.5 mg is prescribed 58.8%, 37.9% is alprazolam 0.25%; and from alprazolam 1 mg is 3.3%. Drug from the group of barbiturates, phenobarbital in measure of 100mg is used 96% vs. 4% application of 15 mg of phenobarbital. Data on consumption of drugs in the related months are important indicators that can be applied to analyze additional epidemiological studies regarding morbidity in certain structures in the population and comparison with data available from other countries.

**Figure 4:**
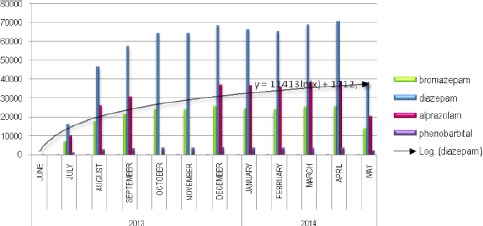
*Overview of realized prescription drugs from BZD group within 12 months*.

Of all the prescriptions and prescribed drugs issued by the group of BZD processed in the database of the Ministry of Health from 2013 when it was introduced, most prescribed BZD for a period of 12 months was 50.7% with diazepam, Alprazolam with 27.7% and 18.8% of Bromazepam prescriptions in this category. Phenobarbiton, the product of a group of barbiturates, has coverage of 1.7% of the prescribed recipes. These data are relevant for comparison between the total issued and implemented prescription drugs registered in the electronic database “My term”, used for the total amount of BZD in the country, thus gaining a clear picture of how drugs are taken with a prescription. Certainly, the data will be complete when the new base will be processing quantities of BZD used internally at a clinics and hospital facilities for the treatment of patients. These data are not included, yet.

According to the Fund [25], the second number of prescription medicines carried by the action of the central nervous system (ATC group “N”), which also have a significant increase in the structure of the total issued drugs from 13% in 2008 to 15% in 2011. The period of analysis 2008-2013, according to the Fund, diazepam is second the most prescribed medicines and alprazolamot is in the tenth place, all of which are medicines authorization for circulation in MKD.

**Figure 5:**
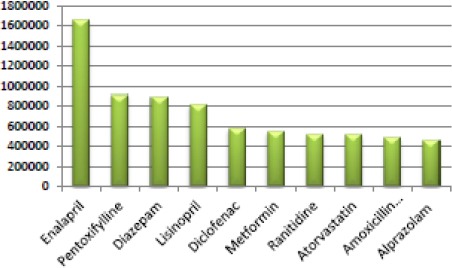
*Top most prescribed drugs in MKD, according to the Health Insurance Fund*.

This review is important to realize that according to available data from Health Insurance Fund database, two drugs from the group of BZD, in the period of 6 years, are the most used drug of all pharmacological groups in comparison with all medical needs.

## Discussion

The aim of this study was to analyze the prevalence of the use of benzodiazepines in the country. The analysis showed a high prevalence of BZD use, especially diazepam. The analysis pointed out the difference in the amounts of data on BZD use in MKD and demand in terms of BZD prescriptions issued. Data on the use for ex. of bromazepam is extremely high, and the number of prescriptions issued alprazolam, indicates that a significant percentage of more substance prescribed by bromazepam. The obtained data indicate lack of prescriptions for medicine prazepam in the pharmacy although the quantities of used prazepam, according to the database are substantial. These data suggests reasonable thinking and considering the use of drugs from the group of BZD without prescription.

If we take into account long analysis of the Fund from 2008 in comparison with the new electronic data base of the Ministry of Health, observed situation with Alprazolam and Bromazepam is the same in the two data bases.

In this context especial concerns are some data from the MKD ESPAD study that confirmed that students and youth less than 9 years old age have access to BZD which is not prescribed by a doctor. In MKD ESPAD study [26], carried out in 1999 and 2008 with coverage of the whole territory of MKD, and in 2012 only city of Skopje was covered. The study of 2008 among other results found that: 10.1% of students had used tranquillizers at least once in life, so they are usually try out illicit substance among students. More girls (11.2%) than students (9.1%) used tranquillizers. For comparison MKD is far above the EU average of 6%. In the same line is using sedatives and tranquillizers only Poland and Lithuania [27]. It should be noted that medical prescriptions for tranquillizers doubled since 1999. Data for 2012 from the ESPAD coverage only city of Skopje suggest that drinking sedatives on the recommendation of a physician 6.6% for less than 3 weeks and 2.5% for more than 3 weeks. 4.5% of students reported using sedatives medical doctor. The age of use of unsubscribed sedatives is 9god or less at 0.87%; 10 years at 0.17%; 11 years. 0.35%; 12 years. 0.70%; 13 years. 0.87%; 14 years. 1.22%; 15 years 2.27%.

Benzodiazepines are a group of CNS depressants, causing a feeling of calm (anxiolysis), drowsiness and sleep, widely used in medicine to treat anxiety (anxiolytics) and insomnia (sedative / hypnotics), and other psychological conditions, such as seizures panic and panic disorder [28], it is widely prescribed BZD drugs in developed countries [29-30]. In France, 30% of people aged 65 years and over use of BZD [31]. They are used by more than 20% of people aged 65 and over in Canada and Spain, and 15% of those in Australia [32-34]. The use of BZD is high among the elderly in the U.S. and the UK [35-36].

European prevalence studies shows that, with the exception of alcohol, benzodiazepines cannabis with psychoactive substances are mostly detected among drivers. Experimental studies show that these drugs impair the ability to drive and when alcohol is used at the same time, the risk of being involved in an accident is greatly increased [37]. EU survey prevalence of the risk of injury in traffic management under the influence of psychoactive means noticed differences between the EU and BZD drugs were mainly found in older female drivers during daylight hours [38]. Epidemiological studies have shown that women are generally more likely to have changes in mood and anxiety disorders than men [39-41].

In conclusion, the analysis in the paper shows that the use of BZD in MKD is particularly high. In the same time, limited number of studies was performed in MKD for this kind of drugs and their effects, still remains unclear which are the drivers for their use; differences in use between the gender; use of BZD by pregnant woman, use of BZD in the adult population. There is need for additional focused research that will contribute to developing a full picture of the situation.
